# The Pathogenesis of Cardiac Arrhythmias in Vitamin D Deficiency

**DOI:** 10.3390/biomedicines10061239

**Published:** 2022-05-26

**Authors:** Maria Barsan, Anca Monica Brata, Abdulrahman Ismaiel, Dinu Iuliu Dumitrascu, Andrei-Vlad Badulescu, Traian Adrian Duse, Stefana Dascalescu, Stefan Lucian Popa, Simona Grad, Lucian Muresan, Carmen Maria Maerescu, Gabriel Cismaru, Vlad Dumitru Brata

**Affiliations:** 1 Department of Occupational Health, “Iuliu Hatieganu” University of Medicine and Pharmacy, 400000 Cluj-Napoca, Romania; maria.opritoiu@umfcluj.ro; 2 Faculty of Environmental Protection, University of Oradea, 410048 Oradea, Romania; 3 2nd Medical Department, “Iuliu Hatieganu” University of Medicine and Pharmacy, 400000 Cluj-Napoca, Romania; abdulrahman.ismaiel@yahoo.com (A.I.); popa.stefan@umfcluj.ro (S.L.P.); costinsimona_m@yahoo.com (S.G.); 4 Department of Anatomy, “Iuliu Hatieganu” University of Medicine and Pharmacy, 400006 Cluj-Napoca, Romania; d.dumitrascu@yahoo.com; 5 Faculty of Medicine, “Iuliu Hatieganu” University of Medicine and Pharmacy, 400000 Cluj-Napoca, Romania; andibadulescu@gmail.com (A.-V.B.); adrianduse@yahoo.com (T.A.D.); dascalescu.stefana@yahoo.com (S.D.); brata_vlad@yahoo.com (V.D.B.); 6 Department of Cardiology, ”Emile Muller” Hospital, 68200 Mulhouse, France; lmure_san@yahoo.com; 7 Department of Obstetrics and Gynecology, “Filantropia” Clinical Municipal Hospital, 200136 Craiova, Romania; mariamaerescu@yahoo.com; 8 Fifth Department of Internal Medicine, Cardiology Rehabilitation, 400347 Cluj-Napoca, Romania; cismaru.gabriel@umfcluj.ro

**Keywords:** arrhythmias, atrial fibrillation, vitamin D deficiency, hypovitaminosis, pathogenesis, sudden cardiac death, ventricular repolarization

## Abstract

The global prevalence of vitamin D deficiency is more than 20%, and the main causes include insufficient intake, reduced absorption, abnormal metabolism, or resistance to its effects. The levels of serum vitamin D appear to influence cardiovascular risk, and the mechanism involved is linked to the transient outward current and the ultrarapid delayed rectifier K+ current densities, activated through the nuclear vitamin D receptor and Akt pathway. A significant number of studies have correlated vitamin D deficiency with an increased risk of developing cardiac arrhythmias and sudden cardiac death. For this reason, the purpose of this review is to analyze the relation between vitamin D deficiency and the pathogenesis of cardiac arrhythmias. Atrial fibrillation, increased QT interval, and QT dispersion were the most common findings associated with vitamin D deficiency. Due to the heterogeneity among existing studies, further research is necessary to confirm the existing data and to analyze its relationship with other types of arrhythmias.

## 1. Introduction

Vitamin D represents a fat-soluble steroid hormone with two main forms: D_2_ and D_3_, ergocalciferol and cholecalciferol, respectively [1]. Exposure to sunlight initiates its synthesis in the skin, but, before it can be functional, it undergoes two processes of hydroxylation: first in the liver to 25(OH)D3 (calcidiol) and then in the kidney to 1,25-dihydroxycholecalciferol (calcitriol)—the active vitamin D hormone [1,2]. This last process is controlled by the parathyroid hormone (PTH) and the serum level of calcium, phosphate, and 1,25(OH)_2_D [1,2]. Vitamin D has many functions, playing an important role in the absorption of calcium and phosphorus, resorption, mineralization and maturation of bone, as well as tubular reabsorption of calcium and its analogues [1]. It can also be used in the treatment of various diseases-like skin conditions, renal osteodystrophy, hypoparathyroidism, cancer, and even COVID-19 [3,4]. Since its synthesis is directly linked to sunlight, it can be influenced by the pigment of the skin, aging, barriers (sunscreen, light passing through glass or plastic; staying indoor, dressing habits), air pollution, latitude, altitude, season, and time of day the sun exposure takes place [1]. The main dietary intake source for vitamin D is fortified food, especially milk [1,5]. Egg yolks, fish oils, butter, and liver are also known natural sources [5].

Insufficient intake, reduced absorption, abnormal metabolism, or resistance to its effects can cause vitamin D deficiency [1,2,5]. The global prevalence of this condition (serum level <30 nmol/L) is more than 20% [5]. A sufficient level amounts to 50–100 nmol/L, and values ≤30–50 nmol/L count as hypovitaminosis [5]. Vitamin D deficiency can cause, depending on age, rickets (children), osteomalacia, and osteoporosis (adults) [5]. The drug categories suspected to affect vitamin D levels include antidepressants, antihypertensives, corticosteroids, chemotherapeutic agents, anti-epileptics, and oral antidiabetics (metformin), among others [6]. Excess serum vitamin D levels (>150–250 ng/mL) are also dangerous with possible outcomes such as gastrointestinal disorders (anorexia, nausea, constipation, or diarrhea), hypercalcemia, hypovolemia, cardiac arrhythmias, suppression of parathyroid hormone, and neuropsychiatric disorders [5].

The levels of serum vitamin D appear to influence cardiovascular risk, with the cardioprotective role of calcitriol proven in murine models [7]. The mechanism involved in ventricular myocytes is an increase in the fast transient outward current and the ultrarapid delayed rectifier K+ current densities, activated through the nuclear vitamin D receptor and Akt pathway [7]. A significant number of studies have correlated vitamin D deficiency with an increased risk of developing heart arrhythmias, arterial hypertension, diabetes mellitus, and sudden cardiac death [8,9].

Lower serum of calcitriol levels are linked to cardiac disease, more specifically associated with increased coronary arteries calcifications, as well as with atrial fibrillation (AF) and heart failure, while downregulation of CYP27B1 (1-hydroxylase) is associated with cardiovascular disease severity [10]. However, single-cell sequencing shows minimal CYP27B1 mRNA expression in heart cells [11], which suggests that the effects of CYP21B1 are mediated by serum vitamin D and are not direct.

For this reason, the purpose of this review is to analyze the relation between vitamin D deficiency and the pathogenesis of cardiac arrhythmias.

## 2. Pathophysiology of Arrhythmias and Conduction Disorders

Data emerging in recent years has strongly linked vitamin D deficiency to cardiovascular disease [12,13]. Aside from its essential role in calcium and bone homeostasis, vitamin D is involved in the physiological processes of various tissues and organs, including the heart [12], via nuclear vitamin D receptor (VDR) activation and transcriptional control of various genes [14]. VDRs were found in cardiomyocytes and cardiac fibroblasts, which are the cells involved in cardiac remodeling processes [15]. Several studies suggested that vitamin D exhibits antioxidant properties and counteracts RAS [16] and other inflammatory pathways [17]. Moreover, a heart-failure (HF) rabbit model by Hanafi et al. showed that the vitamin D metabolite 1,25- dihydroxyvitamin D reduces action potential duration and increases left atrium (LA) contractility, possibly via modulation of calcium release [18].

Furthermore, vitamin D exerts anti-inflammatory effects by counteracting NF-κB signaling in epicardial adipocytes, which can delay the progression of atherosclerosis, coronary remodeling, and ischemic heart disease [19], as well as potentially acting directly on cardiac myeloid cells, which express the vitamin D receptor at a higher level compared to other heart cells [20].

Already established as a vital element in the correct functioning of the cardiovascular system, low vitamin D levels were linked to several diseases, including hypertension [21], peripheral vascular disease [22], coronary artery disease [23], and heart failure [24,25]. In developing hypertension, Forman et al. reported that a low level of vitamin D causes an abnormally active renin–angiotensin–aldosterone system, with an increased aldosterone level and consecutive hypokalemia, increasing susceptibility to developing ventricular arrhythmias [26].

In patients with vitamin D deficiency, the dispersion of the P-wave and the left intra- and inter-atrial electromechanical delay are increased [8]. Paricalcitol restored the levels of myocardial vitamin D receptors and prolonged action potentials in obstructed rats, protecting against myocardial remodeling associated with increased arrhythmogenic risk induced by the decrease in the expression of these receptors [27]. An increased concentration of parathyroid hormone might cause arrhythmias in ischemic heart disease [28]. Low vitamin D levels, poor calcium intake, and renal calcium leak caused by sensitivity to salt and aging generate chronic moderate increases in parathyroid hormone, inducing weight gain, insulin resistance, high blood pressure, left ventricular hypertrophy, and a possible elevation in acute phase reactants [28]. Close monitoring of the parathyroid hormone is of great importance, with decreased levels of the hormone causing hypocalcemia and increased renal phosphate reabsorption [29]. With the parathyroid hormone closely involved in regulating phosphorus levels in the organism, existing evidence suggests that an increased phosphorus level is associated with aortic calcifications, carotid atherosclerosis, and increased ventricular mass, as well as overall increased cardiovascular morbidity and mortality [30,31,32]. Results from the Atherosclerosis Risk in Communities (ARIC) Study have also established a correlation between phosphorus and calcium-phosphorous compound levels and cardiac arrhythmias, with increased levels being associated with a greater risk of AF [33].

Another effect of vitamin D deficiency is the prolongation of the corrected QT interval (QTc) [34]. The implications of vitamin D levels in sudden cardiac death were also investigated: over-supplementation of vitamin D in broilers had a negative outcome, whereas lower 25(OH)D associated with higher parathyroid hormone levels appeared to be independently associated with sudden cardiac death in cardiovascular disease-free older adults [35].

In a series of both in vitro and in vivo studies [36,37,38], vitamin D was also proved to have anticoagulant effects by increasing thrombomodulin expression and reducing tissue factor (TF) expression via VDR on monocytes and endothelial cells [39]. TF is involved in the activation of Factor X and thrombin, both targets of anticoagulant therapeutic agents used to treat AF; therefore, vitamin D may have an influence on LA thrombus formation even under anticoagulant therapy [40].

Despite the availability of an efficient treatment, more than 3.7 million patients die each year worldwide from cardiac arrhythmias [41,42], a number that exceeds the total number of deaths from all cancers in the Western world. Nevertheless, it is still debatable whether the trigger is an asymptomatic clinical profile, a delayed diagnosis, ineffective care, uncertain pathophysiology, or all of the above.

Cardiac arrhythmias present with ambiguous symptoms, from completely asymptomatic to a wide range of symptoms and signs, including palpitations, chest pain, shortness of breath, anxiety, fatigue, lightheadedness or dizziness, blurry vision, and profuse sweating [41,42]. The most frequent symptom of arrhythmia is the sensation of an abnormal heartbeat, called palpitations. Since the risk of myocardial infarction, stroke, sudden cardiac death, syncope, and embolic events is significantly higher in patients with arrhythmias, the clinical profile can be modified by the occurrence of the specific complications, requiring a tailored therapy.

The etiopathogenesis of cardiac arrhythmias is complex, and the most frequent associations include high blood pressure, ischemic heart disease, valvulopathies, congestive heart failure, diabetes, hypoglycemia, hyperthyroidism, electrolyte imbalance, infections, stroke, certain medications, excess of coffee, alcohol abuse, smoking, substance abuse disorder, certain dietary and herbal supplements, psychological stress, and sleep apnea [41,42].

Rare causes of cardiac arrhythmias include fatty acid oxidation disorders, atrial standstill, autoimmune disorders (sarcoidosis, systemic lupus erythematosus, scleroderma, type 1 diabetes, Graves’ disease, rheumatoid arthritis, ankylosing spondylitis, psoriasis, and celiac disease), and genetic conditions called familial arrhythmia (Brugada syndrome, catecholaminergic polymorphic ventricular tachycardia, long QT syndrome, short QT syndrome, Timothy syndrome, Wolff–Parkinson–White syndrome, arrhythmogenic right ventricular dysplasia, idiopathic ventricular fibrillation, and Mahaim syndrome) [43,44].

The complex interacting patterns of ion current activation and inactivation that underpins the action potential production and propagation across successive areas of the heart is fundamentally disrupted in most arrhythmias [45]. Changes in certain genes’ coding-specific ion channels, however, result in well-defined arrhythmic situations, thus providing practical clinical insights into how they could generate arrhythmic tendencies [45].

Changes in the activity of ionic channels or transport systems have a vital role at cellular level. Modifications in electrolyte composition, acidosis or alkalosis, autonomic and hormonal influences, membrane active metabolites, drugs, and poisons are all responsible for these changes. Other factors, such as route shape, anisotropic conduction, and cell coupling resistances, are particularly important in conduction disruptions [46].

## 3. Atrial Fibrillation

Atrial fibrillation is the most common cardiac arrhythmia, affecting an estimated 46.2 million people worldwide in 2016 [47], representing a leading cause of mortality and morbidity, such as thromboembolic stroke and congestive heart failure, even in patients with no prior cardiovascular disease [48,49].

Several mechanisms have been proposed as the explanation of AF development and perpetuation in patients with vitamin D deficit, involving proliferative and proinflammatory actions of renin–angiotensin system (RAS) and catecholamine excess, interstitial fibrosis in the left atrium, and various electrical anomalies that cause fibrillatory conductions [50,51,52,53,54,55,56,57,58]. Both in vivo and in vitro studies exist, which are detailed below.

Although catecholamine excess, activation of RAS, and consequent cascade activation of various neurohumoral agents leading to atrial oxidative stress and inflammation are demonstrated promoters of AF [50,51,52], the precise pathogenesis of AF fibrillation has yet to be determined. AF development relies on an intricate process involving structural and electrical remodeling of the left atrium (LA) [53,54]. Multiple theoretical models involved an increase in atrial interstitial fibrosis as a substrate of AF [55,56,57], as it increases with age [58] and causes electrical conduction abnormalities through the atria, thus creating favorable terrain upon which certain triggers could initiate AF [55,56]. RAS components (including angiotensin II and aldosterone) and profibrotic cytokines, such as transforming growth factor-b1, appear to contribute to these remodeling processes through their proliferative and proinflammatory actions [59]. Although numerous models, both animal [60,61,62] and human [50,58,59], have demonstrated this purported link between interstitial fibrosis and increased AF susceptibility, the mechanisms that generate AF are still under debate [63,64]. Multiple wavelet [57,65], mother rotor [66,67], and focal source [68] mechanisms have all been proposed and demonstrated on animal models; however, a general consensus has yet to be reached.

Considering the associations between vitamin D and AF risk factors and key pathogenetic factors, several recent studies investigated a possible link between low vitamin D levels and AF [40,69,70,71,72,73,74,75]. The main summarized studies investigating the relationship between AF and vitamin D levels are presented in Table 1.

A 2017 matched case-control study by Chan et al. investigated the connection between genetic vitamin D deficiency and AF risk [69]. A cohort of 1175 Chinese cardiac patients underwent genotyping and had their serum 25-hydroxivitamin D measured. Out of twelve single nucleotide polymorphisms (SNPs) investigated, four involved in vitamin D pathways such as vitamin D binding protein (VBP) were associated with low serum 25-hydroxivitamin D [25(OH)D] and strongly correlated with the presence of AF [69]. Measuring life-long exposure to vitamin D by calculating a multi-loci genetic risk score (GRS) for the investigated SNPs helped mitigate the variability of 25(OH)D levels caused by any external factors, which strengthens the link between vitamin D and AF. However, the study still faces a few shortcomings that might limit its generalizability, such as limited ethnic variability and the high-risk nature of the patients, all of whom had stable coronary disease. The design of the study also might enable recall and selection bias to influence the results, but by measuring a genetic exposure to low vitamin D levels, instead of only a baseline vitamin D level, the findings may indeed show a significant link between vitamin D and AF [69].

Canpolat et al. conducted a prospective study comparing LA fibrosis between 48 patients with symptomatic lone paroxysmal AF who underwent cryoballoon-based catheter ablation and 48 healthy subjects using DE-MRI scans [70]. Results showed that serum 25(OH)D levels were significantly lower in patients with lone paroxysmal AF compared to the control group and in patients with moderate-severe LA fibrosis compared to those with mild-moderate LA fibrosis. Moreover, the extent of the LA fibrosis was correlated to AF recurrence during the follow-up period, further confirming the importance of interstitial fibrosis as a mechanism of AF [70].

A recent prospective study by Çakir et al. analyzed the implications of vitamin D levels in the development of thromboembolic complications in patients with AF [40]. A group of 201 patients with AF undergoing non-vitamin K antagonist oral anticoagulant therapy (NOAC) were assigned into two groups based on the presence or absence of a LA thrombus. The study found that plasma levels of 25(OH)D were significantly lower in patients with LA thrombus and AF and independently associated with LA thrombus occurrence; therefore, they may predict thrombotic events in patients with AF despite NOAC treatment [40].

Vitamin D levels in the body are, nevertheless, greatly influenced by several environmental and intrinsic factors, such as lifestyle, diet, seasonal variation, and disease activity [74], which makes it difficult to appreciate the involvement of vitamin D in AF pathogenesis and its therapeutic impact in preventing AF in the general population [73,74]. Meta-analyses by Huang et al. and Zhang et al., respectively [71,72], revealed conflicting results concerning the relationship between vitamin D and the onset of AF, especially when excluding case-control studies. Nonetheless, the variability of individual 25(OH)D levels does not fully counteract the supposed beneficial effects of vitamin D demonstrated in experimental studies. A large retrospective study by Turin et al. investigated the link between vitamin D deficiency and AF in relation to RAS modulation using angiotensin-converting enzyme inhibitors (ACEI) and angiotensin receptor blocker (ARB) drugs [76]. The study concluded that, although there was no significant association between levels of 25(OH)D and AF, findings consistent with previous meta-analyses, vitamin D deficiency appears to attenuate the protective effect of RAS inhibition, thus indicating the need for further investigation of the possible benefits to vitamin D supplementation in specific clinical contexts [76].

A randomized controlled study (RCT) conducted in 2021 by Albert et al. investigated the potential use of vitamin D and marine omega-3 fatty acids in the primary prevention of incident AF [73]. Healthy participants were randomly assigned into groups and received either omega-3 and placebo, vitamin D and placebo, or vitamin D, omega-3 or 2 placebos, over a period of roughly three years and were reevaluated yearly using questionnaires [73]. The trial showed no statistical evidence of an effect of either the omega-3 fatty acid or vitamin D supplementation regarding AF. These findings, therefore, do not support the supplementation of vitamin D as a primary prevention of AF. The large number of participants and the use of questionnaires as a means of data collection may cause an underdetection of AF events, limiting the possibility of observing the smaller effects on AF occurrence [73]. Although large-scale use in AF prophylactic use may not be justified, the experimental benefits of vitamin D supplementation and the demonstrated interaction with the other factors involved in AF mechanisms [76] implies a need for further investigation of possible therapeutic involvement of vitamin D in cardiovascular disease.

In a nationwide Taiwanese study of women treated for osteoporosis with either bisphosphonates or vitamin D, Yang et al. identified a significant increase in AF risk in the bisphosphonate group relative to the untreated control group and a protective effect of vitamin D supplementation relative to controls. Both treated groups had a higher prevalence of hypertension, ischemic heart disease, and cerebrovascular disease compared to controls, with the above-mentioned diseases being risk factors for AF. Subsequently, the protective effects of vitamin D are likely of a greater magnitude than estimated. While the paper is strictly an epidemiological study, the authors mention the beneficial effects of vitamin D supplementation on blood pressure and lipid profile as mediators of its protective effect against AF [20].

In this context, the AF-preventing effects of vitamin D seem even more remarkable considering the link between ischemic heart disease and AF, in that ischemia can induce reentry circuits, ectopic foci, and neuronal remodeling [84]. There is evidence that vitamin D is more directly involved in the above-mentioned ischemia-fibrillation vicious circle, as vitamin D deficiency favors left atrial thrombogenesis in patients with non-valvular AF treated with direct anticoagulants. However, in this study, the left atrial thrombus group had a lower left ventricular ejection fraction and an increased left atrial diameter, which could act as confounding factors [40]. Nonetheless, left atrial enlargement is itself influenced by vitamin D, as will be discussed later. Regarding the mechanisms involved, the authors mention antagonism of renin–angiotensin signaling, as well as the direct effects on the coagulation cascade (upregulation of thrombomodulin), as contributors to the inverse relation between serum calcidiol and atrial thrombus formation [40].

Another observation of the above-mentioned study is that the left atrial thrombus group had, in addition to a significantly lower level of serum vitamin D, a higher C-reactive protein concentration [40]. This is consistent with the anti-inflammatory effects of vitamin D, which counteracts NF-κB signaling in epicardial adipocytes, thereby delaying the progression of atherosclerosis, coronary remodeling, and ischemic heart disease [19], as well as potentially acting directly on cardiac myeloid cells, which express the vitamin D receptor at a higher level compared to other heart cells [11].

Comparing Chinese patients with nonvalvular AF and no other associated cardiovascular condition with healthy controls, patients with a serum calcidiol level below 20 ng/L (threshold for vitamin D deficiency) are twice as likely to have AF compared to those with calcidiol levels over 30 ng/L (threshold for hypovitaminosis). Similar to the study of Çakır et al. [40], the AF group had, in addition to a lower calcidiol concentration, a higher left atrial diameter and an increased serum C-reactive protein [77]. The authors identify left atrial diameter, pulmonary arterial systolic pressure, and C-reactive protein as predictors of calcidiol levels in AF patients, but they do not analyze multicollinearity and do not assess the extent to which these predictors influence one another [77,85].

Consistent with its AF-preventing role [18], the risk of AF after coronary artery bypass grafting (CABG) is significantly increased in vitamin D-deficient patients [78]. Unlike in previous studies, the differences between the postoperative AF and no-AF group regarding C-reactive protein, left atrial diameter, and serum lipids were not significant, thereby strengthening the likelihood of a direct association between serum vitamin D and AF risk [78]. Moreover, the risk of post-CABG AF is approximately halved by preoperative vitamin D supplementation in patients with preexisting vitamin D deficiency [79,80].

However, in the milder situation of vitamin D insufficiency, only Kara and Yasim [79] identified a protective role for vitamin D supplementation, which can be related to the significantly higher doses used by the former (300.000 IU in vitamin D deficiency, 150.000 IU in insufficiency) compared to Cerit et al. (50.000 IU in both groups) [80]. The thresholds for deficiency and insufficiency are consistent between the two papers and the same as those of Chen et al. [77].

Regarding AF risk after any kind of cardiac surgery, a meta-analysis has identified a deleterious effect of vitamin D deficiency [86]. However, the trial of Skuladottir et al. shows contrasting results (i.e., a higher calcitriol level in the AF group), which are, nonetheless, not statistically significant. This outcome can be attributed to confounding factors, in that patients in the AF group are older, less likely to smoke, and have a greater intake of fish [81]. It is noteworthy that the trial differentiates between the two components of calcitriol, namely dihydroxyergocalciferol (of dietary origin) and dihydroxycholecalciferol (of endogenous origin) [81]. The association between postoperative AF and calcitriol is only significant for the former: the fact that it is the minor form of vitamin D in serum, and that it is of dietary origin, further suggests confounding factors as the source of the contradictory results of Skuladottir et al. [81,86].

Additionally, lower serum calcitriol is a risk factor for AF recurrence after cardioversion. However, left atrial diameter is itself positively correlated with AF recurrence risk and negatively with calcitriol levels [82]: while left atrial enlargement is itself a risk factor for AF recurrence after radiofrequency ablation [87], vitamin D supplementation has beneficial effects on left atrial volume, at least in a subset of patients with chronic kidney disease and left ventricular hypertrophy [83]. It is thus unclear whether left atrial enlargement is a confounding or mediating factor.

In the case of catheter ablation, vitamin D deficiency is strongly associated with moderate-severe (Utah III or greater) atrial fibrosis [70], which is itself associated with recurrence [88]. Regarding AF recurrence, serum calcitriol is a highly significant predictor in a univariate Cox regression model but is just above the significance threshold (*p* = 0.053) in a multivariate model. However, these results should be interpreted with care, considering the small sample size (*n* = 48) for a five predictor multivariate regression and the lack of assumption testing [70].

Current evidence exists that correlates low serum vitamin D levels with atrial fibrillation. Nevertheless, further research is needed to better assess the relationship between vitamin D levels and atrial fibrillation given the fact that many of the included studies obtained contradictory results.

## 4. Ventricular Repolarization and Vitamin D Deficiency

Apart from increasing the risk of developing atrial fibrillation, the impact of low vitamin D levels on the dynamic of ventricular repolarization has also been analyzed. More recent research conducted on pediatric patients has also shown a correlation between low vitamin D levels and changes in QT interval, QT interval correction (QTc), QT dispersion (QTd), JT interval, JT interval correction (JTc), Tpeak-to-Tend interval (Tp-e), and Tp-e/QTc [89]. These studies were, more specifically, aimed at the impact of low vitamin D levels on ventricular repolarization and the occurrence of cardiac arrhythmias [90,91].

Bekdas et al. conducted a study on 67 children and adolescents who had similar characteristics and were not suffering from any diseases, dividing them into “Sufficiency”, “Insufficiency,” and “Deficiency” groups, based on serum vitamin D levels [89]. The study investigated the variation of several parameters between the three groups, such as QTc, QTd, JT, JTc, Tp-e, Tp-e/QT, Tp-e/QTc, Tp-e/JT, and Tp-e/JTc. Additionally, the team revealed that patients with insufficient vitamin D levels had a higher pulse, compared to children and adolescents belonging to the “Sufficiency” group (101 ± 18.7 vs. 81.9 ± 13.4) [89]. Nevertheless, significant differences were noticed when comparing the groups with sufficient and deficient vitamin D levels. Thus, Tp-e notably increased in the “Deficiency” group (86.2 ± 10.6 vs. 71.6 ± 6.7), as well as Tp-e/QT, Tp-e/QTc, Tp-e/JT, and Tp-e/JTc [89]. The research also assessed the differences between patients with vitamin D insufficiency and deficiency, concluding that those in the “Deficiency” group had a prolonged Tp-e (86.2 ± 10.6 vs. 74.4 ± 9.2), a lower QTc (402.6 ± 21.1 vs. 426.3 ± 25.1) and, consequently, a higher Tp-e/QTc ratio (0.21 ± 0.02 vs. 0.17 ± 0.01) [89]. However, the study found no considerable distinctions in the values of certain measured parameters between the “Sufficiency” and “Deficiency” groups, such as QTc and JT (*p* = 0.25 and *p* = 0.75, respectively) [89].

Bagrul and Atik conducted a similar study, including 150 adolescents, in which they measured their vitamin D levels and organized them into vitamin D “Sufficient”, “Insufficient,” and “Deficient” [90]. Moreover, QTc, QT dispersion (QTd), JT dispersion (JTd), Tp-e, Tp-e/JTpeak, and Tp-e/QTc were measured [90]. The study concluded that adolescents with deficient and insufficient vitamin D levels had a prolonged Tp-e, when compared to patients from the “Sufficient” group. Consequently, these patients had an increased Tp-e/JTpeak ratio. Similarly, patients with vitamin D deficiency had a significantly increased JTd when compared to both other groups, as well as a higher QTd [90]. Additionally, the study also recorded an increased Tp-e/QTc ratio in patients with deficient and insufficient vitamin D levels [90].

Another study on patients with type 2 diabetes investigated the relationship between 25-hydroxyvitamin D deficiency and QT interval duration and dispersion [91]. Yetkin et al. included 253 diabetic patients and 170 healthy controls without prior cardiovascular disease or known history of cardiac arrhythmias and assessed the frequency of vitamin D deficiency in the two groups, as well as prolonged QT intervals. The study concluded that diabetic patients with prolonged QTc and a higher QTc dispersion were more frequently vitamin D deficient [91]. Thus, 65.4% of the patients with type 2 diabetes were suffering from a vitamin D deficiency, as well as 64.7% of the total volunteers. However, a prolonged QTc interval was observed in 26.8% of the diabetic patients, in comparison with only 4.1% in the healthy group. Nevertheless, advanced age, higher HbA1c levels, and a longer disease duration were also less strongly associated with increased QTc intervals [91]. The main studies assessing the relationship between vitamin D levels and ventricular repolarization are summarized in Table 2.

There is a significant correlation between vitamin D deficiency and disturbances in ventricular repolarization; numerous studies show notable differences between QT interval durations in patients with normal or low vitamin D levels [89,90,91]. Although the relationship between vitamin D and heart arrhythmias is established, further research is needed in order to better understand the impact that vitamin D has on the pathogenesis of cardiac arrhythmias.

## 5. Conclusions

The occurrence of atrial arrhythmias, mainly atrial fibrillation, together with significant changes in ventricular repolarization, represented by a prolonged QTc interval, are positively associated with vitamin D deficiency. Due to the heterogeneity among existing studies, further research is necessary to confirm the existing data and to analyze other types of arrhythmias.

## Figures and Tables

**Table 1:** The relationship between vitamin D and AF risk factors.

Author (Year)	Study Design and Participants’ Characteristics	Parameters Investigated	Outcome
Çakır et al. [40] (2020)	observational study 201 patients suffering from AF (133 female) following treatment with continuous non-vitamin K antagonist oral anticoagulant	thrombus occurrence	low 25(OH)D levels associated with dense spontaneous echo contrast and LA thrombus occurrence
Chan et al. [69] (2017)	case-control study 156 patients with AF 1019 control all female	SNPs of vitamin D mechanistic pathways and 25(OH)D levels in serum	genetically deprived vitamin D exposure constitutes a predisposition for a higher risk of AF in patients with coronary artery disease
Canpolat et al. [70] (2017)	prospective study 48 patients (41.7% female) suffering from lone paroxysmal AF 48 healthy controls	LA fibrosis	lower 25(OH)D levels are significantly linked to more considerable LA fibrosis and may play a role in relapse after cryoablation
Albert et al. [73] (2021)	randomized clinical trial 25,119 patients without preexisting cardiovascular disease (incl. AF) and cancer; aged 50 and higher	6272 subjects: 460 mg/d eicosapentaenoic acid + 380 mg/d of docosahexaenoic acid + 2000 IU/d vitamin D3 6270 subjects: eicosapentaenoic and docosahexaenoic acid + placebo 6281 subjects: vitamin D3 + placebo 6296 subjects: 2 placebos	over a median follow-up of 5 years, no significant differences in the occurrence of AF
Turin et al. [76] (2018)	retrospective study 47,062 patients with documented 25(OH)D levels	incidence of AF in patients with ACEI −/+ ARB treatment vs. patients not following treatment with ACEI or ARB	use of ACEI/ARB links to less AF events (attenuated in patients taking 25(OH)D) vitamin D deficiency not statistically significant associated with AF incident
Yang et al. [20] (2018)	observational study 20,788 female patients diagnosed with osteoporosis	implication of osteoporosis treatment in the occurrence of AF	different risk for AF associated with diverse osteoporosis treatment; vitamin D could have beneficial effects in patients suffering from osteoporosis
Chen et al. [77] (2014)	observational study 162 patients with nonvalvular persistent AF; without any other cardiovascular disease 160 healthy controls	25(OH)D serum levels	low vitamin D levels associated with AF occurrence in Chinese adults with no other vascular risk factors
Özsin et al. [78] (2018)	prospective randomized clinical trial 50 patients with postoperative atrial fibrillation (66% male) 50 patients without postoperative atrial fibrillation (74% male)	AF occurrence until discharge, immediate measurement of 25(OH)D serum levels after the event	lower levels of 25(OH)D could be one of the reasons for postoperative atrial fibrillation and are an independent predictor for this event
Kara and Yasim [79] (2020)	randomized controlled, blinded, and parallel-arm trial 116 patients with vitamin D deficiency or insufficiency who had coronary artery bypass grafting: 58 patients with oral vitamin D supplementation 48 h before procedure = treatment group and 58 patients without any vitamin D supplementation = control	occurrence of postoperative atrial fibrillation until discharge	significant prevention of postoperative atrial fibrillation with short-term preoperative supplementation of vitamin D
Cerit et al. [80] (2018)	randomized, blinded clinical trial 328 consecutive patients with on-pump coronary artery bypass grafting 80 patients with vitamin D insufficiency and 56 patients with vitamin D deficiency; treatment group: 68 patients with oral vitamin D 48 h before surgery; control group: 68 patients without oral vitamin D	occurrence of postoperative atrial fibrillation until discharge	preoperative vitamin D supplementation strongly associated with the prevention of occurrence of postoperative atrial fibrillation in patients suffering from vitamin D deficiency
Skuladottir et al. [81] (2016)	randomized, double-blind, placebo-controlled clinical trial 118 patients undergoing coronary artery bypass grafting and/or valvular repair surgery with available preoperatively and postoperatively (the third day after) plasma samples of vitamin D2 and vitamin D3	occurrence of postoperative atrial fibrillation	no association for plasma levels of total 25(OH)D and 25(OH)D_3_; higher levels of 25(OH)D_2_ linked with higher occurrence of postoperative atrial fibrillation
Yaman et al. [82] (2020)	retrospective study 52 patients with AF and rhythm control strategy scheduled for medical or electrical cardioversion	recurrence of atrial fibrillation after cardioversion and vitamin D levels	increased risk of AF recurrence associated with lower vitamin D levels
Tamez et al. [83] (2012)	randomized trial 196 patients suffering from chronic kidney disease, left ventricular hypertrophy (mild to moderate) with preserved ejection fraction receiving either 2 μg of oral paricalcitol or placebo for 48 weeks	two-dimensional echocardiography and levels of brain natriuretic peptide	patients receiving an analogue of vitamin D presented reduced left atrial volume index and an attenuated rise in brain natriuretic peptide

**Table 2:** The relationship between vitamin D levels and ventricular repolarization.

Author (Year)	Study Design and Participants’ Characteristics	Parameters Investigated	Outcome
Bekdas et al. [89] (2021)	Observational study 67 children and adolescents with the following vitamin D levels: - sufficient: 44 - insufficient: 13 - deficient: 10	QRS QTmin,max,av Pulse QTc,d,dc JT, JTc Tp-e Tp-e/QT Tp-e/QTc Tp-e/JT Tp-e/JTc	Vitamin D deficient: prolonged Tp-e, Tp-e/QT, Tp-e/QTc, Tp-e/JT, and Tp-e/JTc. Vitamin D insufficient: lower QTmax and QTmin, increased pulse, and JTc.
Bagrul and Atik [90] (2019)	Observational study 150 adolescents with the following vitamin D levels: - sufficient: 50 - insufficient: 50 - deficient: 50	QT QTc,d Tp-e Tp-e/QTc JT JTd Tp-e/JTpreak	Vitamin D deficient: prolonged Tp-e, increased Tp-e/QTc ratio, increased Tp-e/JTpeak ratio, higher QTd, and JTd Vitamin D insufficient: prolonged Tp-e, increased Tp-e/QTc ratio, increased Tp-e/JTpeak ratio, lower QTd, and JTd when compared to the “Deficiency group”.
Yetkin et al. [91] (2015)	Observational study 423 patients among which: - 253 patients with type 2 diabetes - 170 healthy volunteers	QTc QTd HbA1c	QTc and QTd prolongation were associated with type 2 diabetes mellitus, advanced age, a longer duration of the disease, and higher HbA1c levels.

## Data Availability

Not applicable.
